# Immune contexture defined by single cell technology for prognosis prediction and immunotherapy guidance in cancer

**DOI:** 10.1186/s40880-019-0365-9

**Published:** 2019-04-18

**Authors:** Tong Wu, Xuan Wu, Hong-Yang Wang, Lei Chen

**Affiliations:** 1 International Cooperation Laboratory on Signal Transduction, Eastern Hepatobiliary Surgery Institute, Second Military Medical University, Shanghai, 200438 P. R. China; 2 National Center for Liver Cancer, Shanghai, 201805 P. R. China; 3 Central Laboratory, Shanghai Tenth People’s Hospital, Tongji University, Shanghai, 200070 P. R. China; 4 Department of Laboratory Medicine, Shanghai Tenth People’s Hospital, Tongji University, Shanghai, 200070 P. R. China

**Keywords:** Tumor microenvironment, Single cell technology, Immune contexture, Tumor infiltrating leukocytes, Prognosis, Immunotherapy

## Abstract

Tumor immune microenvironment is closely related to tumor initiation, prognosis, and response to immunotherapy. The immune landscapes, number of infiltrating immune cells, and the localization of lymphocytes in the tumor vary in across different types of tumors. The immune contexture in cancer, which is determined by the density, composition, functional state and organization of the leukocyte infiltrate of the tumor, can yield information relevant to the prediction of treatment response and patients’ prognosis. Better understanding of the immune atlas in human tumors have been achieved with the development and application of single-cell analysis technology, which has provided a reference for prognosis, and insights on new targets for immunotherapy. In this review, we summarized the different characteristics of immune contexture in cancer defined by a variety of single-cell techniques, which have enhanced our understanding on the pathophysiology of the tumor microenvironment. We believe that there are much more to be uncovered in this rapidly developing field of medicine, and they will predict the prognosis of cancer patients and guide the rational design of immunotherapies for success in cancer eradication.

## Introduction

Tumor microenvironment (TME) is the cellular environment in which tumor cells reside. It is composed of various stromal cell types, including immune and inflammatory cells, adipocytes, fibroblasts, vascular endothelial cells, which are surrounded by intercellular interstitial, microvascular and infiltrating molecules. In the past, the understanding of tumor heterogeneity was mainly focused on tumor cells. Cancer-associated stromal cells including immune cells and fibroblasts in the TME have been identified to be highly heterogeneous in recent studies [1, 2]. Among them, the T cells, B cells, natural killer (NK) cells, and other types of lymphocytes, which also have important roles in the tumor immune microenvironment (TIME), have been the main research hotspots in recent years [1, 2].

Tumor immune contexture refers to the spatial organization and density of the immune infiltrate in the TME [3]. TIME is usually associated with the clinical outcome of cancer patients, and has been used for estimating cancer prognosis [3]. For instance, the infiltration of large numbers of cluster of differentiation 8 positive (CD8^+^) cytotoxic T cells, type 1 T helper (Th1) cells, and associated cytokines in TME usually indicate that the immune system can inhibit tumors to some extent, suggesting the existence of a robust antitumor milieu that can lead to eradication of tumors [4]. Therefore, researchers have uncovered potentially targeted features of the tumor immune contexture, among which the programmed cell death ligand-1/programmed cell death protein-1 (PD-L1/PD-1) axis have been particularly attractive [5].

The highlight of the single cell analysis technique is the use of multiple parameters to analyze individual cells, which can reveal the heterogeneity and homogeneity of cells. In the emerging single cell protein detection technologies, mass cytometry is the most representative one, as it can detect dozens of proteins on a single cell simultaneously [6, 7]. In addition, the next-generation sequencing technology including single cell genomics and single cell transcriptomics made it possible to identify and characterize the cell types in heterogeneous tissues [8]. Both the heterogeneity of cells in one tumor sample and the different characteristics of immune contexture between distinct tumor samples can reflect the heterogeneity of clinical samples. Single cell analysis can also be very convenient for comparing samples from different cancer patients to find specific differences in tumor immune contexture. Better understanding on the pathophysiology of the tumor microenvironment by single cell technology will predict the prognosis of cancer patients and guide the rational design of immunotherapies for success in cancer eradication. These data can be used as an important basis for individualized treatment. In this review, we summarize the diverse immune contexture in several types of tumors revealed by single cell analysis technology, and provide new strategies for prognosis prediction and immunotherapy guidance in cancer.

## Respiratory tumor

### Immune contexture

Small cell lung cancer and non-small cell lung cancer (NSCLC) are the two main histological types of lung cancer. NSCLC accounts for 85% of lung cancers and used to be subdivided into lung squamous cell carcinoma and adenocarcinoma [9–11]. In lung cancer, greater focus has been placed on tumor-infiltrating lymphocytes (TILs) as they have been found to be able to directly affect prognosis and the response to immunotherapy [12–14]. The TIME of lung cancer is mainly composed of T cells, macrophages, and mast cells [11, 15–17]. In NSCLC, the number of CD8^+^ cells, CD4^+^ cells, T cells, and B cells are increased in tumor tissues as compared to normal lung tissues [18], among which the increase of B cells was found to be the most distinct [9]. Recently, Lavin et al. [19] have found that there are notable alterations of T cells and NK cells in lung adenocarcinoma. Moreover, considerable changes in tumor-infiltrating myeloid (TIM) cells have been identified to weaken the T cells-mediated anti-tumor immunity and promote tumor progression [20, 21]. Dendritic cells (DCs) were found to be the most important components among TIM to induce the activation of T cells [22, 23], and CD103^+^ DCs were shown to drive CD8^+^ T cells activation [23–26]. Compared with the patients with high CD8^+^ T cells but low DCs density in their tumor mass, NSCLC patients with high level of both CD8^+^ T cells and DCs had significantly better survival rate [27]. It has been found early lung adenocarcinoma lesions have significantly reduced NK cell compartments [19, 28]. Furthermore, changes in TIM subsets may compromise anti-tumor T cell immunity [19, 28]. Regulatory T cells (Tregs) with highly expressed markers including CD39, cytotoxic T lymphocyte antigen 4 (CTLA-4), CD137, and inducible T cell costimulatory (ICOS) were found observably increased in lung tumor [19]. Besides, macrophages in lung tumors also had unique characteristics compared with those in normal lung tissues, which were characterized by high-level expression of CD64, peroxisome proliferator-activated receptor γ (PPARγ), CD14, CD11c and low-level expression of CD86 and CD206 [19].

### The role of TILs

TILs are T cell populations with higher specific immune response to tumor cells than non-infiltrating lymphocytes [29, 30]. TILs are divided into three subtypes: epithelial lymphocytes within the cancer cell nests, stromal lymphocytes in the central cancer stroma, and peritumoral lymphocytes along the invasive margin [31]. The role of TILs in promoting or inhibiting tumor progression has not yet been determined [32]. Generally, TILs are closely related to the clinical outcomes of cancer [33]. The subset, location, and density of TILs can be used to predict the overall survival (OS) and progression-free survival (PFS) of lung cancer patients, using immunoscore strategy [34, 35]. These cells infiltrated in tumor epithelium or stroma play an active role in inhibiting the progression and metastasis of tumors. Studies have found that high infiltration of CD3^+^ cells in tumor epithelium and stroma suggest a better OS, while high infiltration of forkhead box P3 positive (FOXP3^+^) T cells in tumors was associated with poor prognosis [36]. Fas/Fas ligand (Fas/FasL) pathway disorder has been reported to induce malignant lung cells to evade the killing effect of cytotoxic T cells and thus resist the immune system [37, 38]. Recent studies have shown that NSCLC patients with low ratio of CD4^+^/CD8^+^ in peripheral blood T cells may have better prognosis [39]. The response of NSCLC patients to platinum neoadjuvant chemotherapy can be predicted by the density of CD8^+^ and FOXP3^+^ TILs in TIME [40]. In addition, the expression levels of inhibitory receptors such as T-cell immunoglobulin and mucin-domain containing-3 (TIM-3), lymphocyte-activation gene 3 (LAG-3), PD-1, and CTLA-4 in CD4^+^ or CD8^+^ T cells can be applied to predict the response to lung cancer immunotherapy [40–42].

### Immunotherapy

In the past decades, chemotherapy has been regarded as the standard treatment for lung cancer, but the therapeutic effect has been limited. The emergence and clinical application of immunotherapy have brought hope to countless patients by improving their PFS and OS rate. Tumor cells can suppress the immune system by activating immune checkpoints to achieve immune escape [43]. Among the immune checkpoints, PD-1 and CTLA-4 expressed by T cells and PD-L1 expressed by tumor cells have been the most studied. Immune checkpoint inhibitors are antibodies interacting with these immune checkpoint proteins to relieve immunosuppression and they have been used in patients with NSCLC. Studies have found several factors that can affect the response to immunological checkpoint therapy including tumor antigens (e.g. neoantigens, tumor mutation burden, microsatellite instability) [44], immunosuppressive and inflammatory cells or proteins (e.g. TILs and tumor-associated immune cells, gene signatures, CTLA-4, IDO, PD-L1) [45], and host factors (single-nucleotide polymorphisms and microbiome) [46]. These indicators can be used to predict the effectiveness of immunotherapy for patients. Clinical trials of anti-PD-1 or anti-PD-L1 combined with anti-CTLA-4 drugs for NSCLC have been carried out, and positive results have been preliminarily obtained which mainly reflected in a higher OS rate and durable responses [47].

## Digestive system tumors

### Immune contexture

Single cell technology can also be applied to analyze the immune microenvironment of the digestive system. Zheng et al. [48] used single-cell sequencing to analyze T cell populations and depicted a distinctive immune landscape of liver cancer. They found fewer CD8^+^ T cells in the center of the tumor than in the outer cortex. The proportion of CD4^+^ CD25^high^ T cells in tumors is higher than that in normal tissues or peripheral blood, which can confirm that Tregs are enriched in the TME. Unlike Tregs, mucosa-associated invariant T cells (MAITs) are enriched in non-neoplastic adjacent liver tissues, while effector memory T cells (TEMs) and primitive T cells expressing C-X3-C motif chemokine receptor 1 (CX3CR1) and naive T cells expressing CC-chemokine receptor 7 (CCR7) are enriched in peripheral blood [48]. The percentage of exhausted CD8^+^ T cells in tumor specimens is significantly higher in advanced patients, which indicated that the TME of hepatocellular carcinoma (HCC) could induce the transformation of CD8^+^ infiltrating T cells into an exhausted state [49]. High expression of exhaustion-related genes including T-cell immunoreceptor with Ig and ITIM domain (TIGIT) and CTLA-4 was also found in tumor-infiltrating Tregs [49]. CD4^+^T cells were also analyzed, and independent developmental pathways of infiltrating CD4^+^ T helper cells in tumors were identified. Xiao et al. [50] identified a new B cell subset with a high expression of carcinogenic PD-1 in HCC. This subset had a unique phenotype of CD5^high^ CD24^−/+^ CD27^high/+^ CD38^dim^, which was different from the traditional peripheral regulatory B cell phenotype of CD24^high^ CD38^high^.

As one of the leading causes of cancer-related death worldwide, gastric cancer is a serious threat to human health [51]. FOXP3^+^ Tregs are abundant in gastric cancer tissues, and the high infiltration of FOXP3^+^ Tregs in tumors may indicate a better prognosis of gastric cancer [52]. It has been found that patients with a high expression level of CD8^+^ and FOXP3^+^ TILs in epithelium have better clinical outcomes [53]. The short distance between CD8^+^ and FOXP3^+^ cells has been hypothesized to be related to poor prognosis, as tumor-specific CD8^+^ T immune responses were suppressed by Tregs [53]. Okita et al. [54] have demonstrated that the level of CD11b^+^ antigen presenting cells (APC), neutrophils and Tregs are increased in metastatic tumor-draining lymph node (MTDLN), while the proportion of T effector cells (TEs) and TEMs was significantly lower in MTDLN. The long-term protective effect of CD4^+^ TEM against tumor has been confirmed. Tolerant semi-mature CD11b^+^ PD-L1^+^ APCs stimulated by gastric cancer cells can not only reduce TE and TEM but also induce Tregs proliferation [54].

### The role of TILs

The balance of cytotoxic T cells and Tregs in tumors has been found to be an independent predictor for survival and recurrence in HCC, and Tregs affect the invasiveness of HCC [54]. High activated CD8^+^ cytotoxic lymphocytes (CTLs) in combination with low Tregs in tumor can improve both OS and disease-free survival (DFS) of patients, while CD4^+^ TILs, CD8^+^ TILs or CD3^+^ TILs had no effect on patients survival. In addition to the above effects, high-density infiltration of Tregs is also related to tumor vascular invasion and the lack of tumor cysts [55].

### Immunotherapy

In the field of liver cancer, immunological checkpoint treatment is mainly focused on CTLA-4 and PD-L1/PD-1 pathways [56]. Clinical trials of blocking CTLA-4 with tremelimumab to reactivate immune response in the treatment of advanced HCC have shown remarkable efficacy [43]. Blocking PD-1 with nivolumab to stimulate immune responses in the treatment of patients intolerance to sorafenib treatment or patients with refractory HCC has been found effective [56].

## Breast tumor

### Immune contexture

Compared with normal tissues, breast tumors have more abundant cell populations and greater phenotypic heterogeneity [57, 58]. Azizi et al. [59] identified that unlike the M1/M2 macrophage polarization model in breast tumor, T cell activation was a continuous process. They found that B cells were enriched in lymph nodes, while naive T cells were concentrated in peripheral blood. More cytotoxic T cells, activated macrophage clusters, and Tregs accumulated in tumors rather than in non-tumorous tissues or peripheral blood. Increased gene expression variance in tumor compared to normal tissue leads to significantly increased diversity of cell states. Under varied environmental conditions, the gene expression of Treg clusters did not show significant heterogeneity, while significant differences in exhaustion, hypoxia and anti-inflammatory gene signature of TEs were observed [59]. The traditional view in macrophages is that the activated states of M1 and M2 are mutually exclusive [60]. However, recent research outcomes challenge this view. It has been found that both pro-inflammatory M1 and tissue reparative M2 genes can be expressed simultaneously in the same macrophage and they may be interdependent [61].

### The role of TILs

In breast cancer, TILs can indicate the response to chemotherapy or immunotherapy and are associated with patient survival [62–64]. PD-1 expression in T cells involved in tumors often associates with a state of T cell exhaustion. However, CD8^+^ TILs with PD-1 expression in human breast tumor retain strong capacity for degranulation and cytokine production [65, 66]. Different from the role of CD8^+^ TILs in breast tumor which is associated with improved clinical outcomes, CD4^+^ TILs including FOXP3^+^ Tregs, Th1, and Th17 cells have controversial prognostic roles. Some studies have shown that patients with high levels of FOXP3^+^ TILs have poor chemosensitivity and worse prognosis, while others reported that breast cancer patients with high levels of FOXP3^+^ T cells had better outcomes [67, 68].

### Immunotherapy

Breast tumors can be divided into “inflamed” tumors and “non-inflamed” tumors according to the level of interferon (IFN)-producing and cytotoxic TILs, or expression level of PD-L1 in tumors and PD-1 in immune cells [69]. Immune checkpoint therapy harvests the best efficacy in “inflamed” tumors which are characterized by high level of IFN-producing and cytotoxic TILs, or high-level expression of PD-L1 in tumors and PD-1 in immune cells [69]. Few breast tumors are considered to be “inflamed” tumors. Triple-negative breast cancer (TNBC) is usually viewed as the most “inflamed” breast cancer subtype, so most immunotherapy trials for breast cancer, including immunosuppressive agents against PD-1 or PD-L1, such as atezolizumab, have been applied to TNBC [70]. However, for “non-inflamed” breast tumors such as those being estrogen receptor positive (ER^+^) or human epidermal receptor 2 negative (HER2^−^) breast tumors, improving the efficacy of immunotherapy remains a challenge [71].

## Immune contexture in other tumors

### Clear cell renal cell carcinoma (ccRCC)

Chevrier et al. [72] depicted a landscape of TME in ccRCC patients using single-cell analysis. Various biomarkers expressed by T cells and tumor-associated macrophages (TAMs) can be used as potential targets of immunotherapy [73]. CD38, the most overlapping marker with PD-1, has been found as a potential marker of T cell depletion in ccRCC [74]. Macrophages in the TME can co-express anti-tumor marker (CD169) and pro-tumor markers (CD163, CD204, and CD206) [75]. ccRCC has the highest heterogeneity of TAMs superior to CD8 composition and CD4 composition [72]. It has been identified that ccRCC TAMs have a unique M2 phenotype which can induce Tregs amplification, characterized by the expression of IL-10, CD163, fibronectin 1, and IFN-4 [76–78].

### Hodgkin’s lymphoma (HL)

The most common subtype of malignant lymphoma in young people is Hodgkin’s lymphoma (HL) [79]. Studies have focused on the TME of classical HL with the use of mass cytometry analysis. In some human solid tumors, the blocking effect of PD-1 is related to the activation of CD8^+^ cytotoxic T cells in TME [80–85]. In most classical HLs, the simultaneous increase of CD4^+^ Th1 polarized Tregs and differentiated T effectors were observed [86]. The complementary mechanism may exist in the immunosuppression of classical HL, which made the expression level of PD-1 in CD4^+^ Th1 polarized T effectors and Tregs differ greatly [86]. PD-1^+^ T cells in peripheral blood of HL patients were found increased compared with healthy people and several Phase I dose escalation trials showed effective treatment of nivolumab (anti-PD-1 antibody) [87].

### Melanoma

Melanoma can be divided into four subtypes according to the mutation genotypes. They are BRAF, N-Ras, K-Ras, and H-Ras-mutant melanomas [88]. Single-cell RNA-seq has been applied to study the composition of tumor immune cells in melanoma. Both CD4^+^ T cells and CD8^+^ T cells have different transcriptional states in tumors, which shows a continuous transition from an early effector to a dysfunctional T cell state [89]. Dysfunctional CD8^+^ T cells characterized by high expression of immune checkpoint molecules, such as LAG-3 and PD-1, are positively correlated with tumor malignancy [90]. It has been found that TCF7^+^ CD8^+^ T cells can predict positive clinical outcomes of patients treated with anti-PD-1 antibody [91].

Single cell-analytic methods used in some other types of tumors are detailed in Table 1.

**Table 1:** Single cell-analytic methods used in different cancers

Cancer	Single cell-analysis technology	References
Melanoma	Single-cell RNA sequencing	[100–102]
	Single-cell barcode chip (SCBC)	[103]
Leukemia	Single-cell RNA sequencing	[104]
	Single-cell exome sequencing	[105]
	Single-cell mass cytometry	[106]
Pancreatic cancer	Single-cell RNA sequencing	[107, 108]
Cervical carcinoma	High-dimensional single-cell mass cytometry	[109–111]
	Single-cell whole-genome sequencing	[75, 111]
Glioma	Single-cell RNA sequencing	[112–115]
Thyroid carcinoma	Single-cell DNase sequencing (scDNase-seq)	[116, 117]

## Single-cell technology

### Single-cell mass cytometry

Traditional flow cytometry can only detect less than a dozen protein markers, making it difficult to analyze small numbers of cells or complex phenotypes [92]. Mass flow cytometry removes the limitations of flow cytometry by using flow cytometry in conjunction with mass spectrometry to significantly increase the number of detectable labels [93]. Mass cytometry is a sensitive measurement method based on inductively coupled plasma mass spectrometry. Samples with the antibody-metal coupling labels are vaporized and ionized in argon plasma, after which the detector determines the elemental composition of a sample by measuring the time-of-flight (ToF) of the ionized elements.

### Single-cell RNA sequencing (scRNA-seq)

Single-cell RNA sequencing (scRNA-seq) can identify and characterize the cell types in heterogeneous tissues and different conditions via transcriptomic measurements at single cell resolution [94]. Different from the first scRNA-seq experiment in 2009 which profiled only eight cells [95], we now can obtain a large amount of scRNA-seq data to provide detailed biological information of the cells found in a sample [96]. scRNA-seq method can analyze thousands of single cells in a single experiment to form gene expression profiles which is sufficient to classify cell types. Although scRNA-seq provides detailed transcriptional catalogues of individual cells, we still need to extract more focused gene expression information from it.

### Single-cell proteomics

As the main undertaker of life activities, the protein components of cells can indicate biological processes and diseases. Novel single-molecule protein sequencing schemes that use tunneling currents, fluorescence and nanopores realize single-cell proteomics and greatly improve the sensitivity of low abundance protein detection [97]. Mass spectrometry can use tens of thousands of cells or more to detect and quantify proteomes [98]. Some researchers analyzed the subcellular distribution of more than 12,000 human proteins using antibody-based immunofluorescence confocal microscopy [99]. By fluorescently labeling selected amino acids, an old method for protein sequencing called Edman degradation can be improved [98].

The characteristics of several single cell-analytic methods are detailed in Table 2.

**Table 2:** Advantages and disadvantages of several single cell-analytic methods

Single cell analysis technology	Advantages	Disadvantages	References
Single-cell mass cytometry	Detect multiple protein markers simultaneously	Providing only a coarse level of resolution	[92, 93]
Single-cell RNA sequencing	Characterize the transcriptomes of cell types in different tissues and identifying their transcriptional signatures	Initial amounts of RNA obtained from a single cell are low Exhibiting higher levels of noise and more zero values	[94, 96]
Single-cell proteomics	Capture proteomic information from individual cells Rich in information	Multicellular Single-cell proteomics with high sensitivity, specificity and low cost needs to be developed	[97–99]

## Conclusions

The immune cells in TME can be divided into different clusters according to their surface markers and functions, and within the clusters of lymphocytes, there is extensive heterogeneity among different tumors. The immunological profiles of different tumors were recently revealed by single-cell analysis technology including mass cytometry and single cell sequencing. Analysis of the composition of TME improves the ability to predict the prognosis of different cancers, thus provides new strategies for cancer treatment.

Determining the characteristics and differences of different TIME is necessary for tumor immunotherapy. In order to effectively predict patients’ response to immunotherapy and reveal new therapeutic targets, higher resolution techniques are waiting to be applied to assess the composition, functional status and cellular localization of total immune cells in TIME.

## Abbreviations

TME: tumor microenvironment
NK: natural killer
TIME: tumor immune microenvironment
CD: cluster of differentiation
Th1: type 1 T helper
PD-L1: programmed cell death ligand-1
PD-1: programmed cell death protein-1
TIL: tumor infiltrating lymphocyte
TIM: tumor-infiltrating myeloid
DC: dendritic cell
Treg: regulatory T cell
CTLA-4: cytotoxic T lymphocyte antigen 4
OS: overall survival
PFS: progression-free survival
FOXP3: forkhead box P3
MAIT: mucosa-associated invariant T cell
TEM: effector memory T cell
HCC: hepatocellular carcinoma
APC: antigen presenting cell
MTDLN: metastatic tumor-draining lymph node
TE: effector T cell
CTL: cytotoxic lymphocyte
DFS: disease-free survival
TNBC: triple-negative breast cancer
ccRCC: clear cell renal cell carcinoma
TAM: tumor-associated macrophage
IFN: interferon
HL: Hodgkin’s lymphoma
LAG-3: lymphocyte-activation gene 3
scRNA-seq: single-cell RNA sequencing
